# Latest insights into the epidemiology, characteristics, and therapeutic strategies of chronic hepatitis B patients in indeterminate phase

**DOI:** 10.1186/s40001-024-01942-0

**Published:** 2024-06-21

**Authors:** Junye Liu, Yan Yu, Heping Zhao, Lei Guo, Wenjuan Yang, Yuzhu Yan, Jing Lv

**Affiliations:** 1 Department of Clinical Laboratory, Honghui Hospital, Xi’an Jiaotong University, Youyi Dong Road, Xi’an, 710054 China; 2 Department of Spinal Surgery, Honghui Hospital, Xi’an Jiaotong University, Youyi Dong Road, Xi’an, 710054 China

**Keywords:** Antiviral therapy, Chronic hepatitis B, Epidemiology, Hepatitis B virus, Immune phase, Indeterminate phase

## Abstract

As a hepatotropic virus, hepatitis B virus (HBV) can establish a persistent chronic infection in the liver, termed, chronic hepatitis B (CHB), which causes a series of liver-related complications, including fibrosis, cirrhosis, and hepatocellular carcinoma (HCC). HCC with HBV infection has a significantly increased morbidity and mortality, whereas it could be preventable. The current goal of antiviral therapy for HBV infection is to decrease CHB-related morbidity and mortality, and achieve sustained suppression of virus replication, which is known as a functional or immunological cure. The natural history of chronic HBV infection includes four immune phases: the immune-tolerant phase, immune-active phase, inactive phase, and reactivation phase. However, many CHB patients do not fit into any of these defined phases and are regarded as indeterminate. A large proportion of indeterminate patients are only treated with dynamic monitoring rather than recommended antiviral therapy, mainly due to the lack of definite guidelines. However, many of these patients may gradually have significant liver histopathological changes during disease progression. Recent studies have focused on the prevalence, progression, and carcinogenicity of indeterminate CHB, and more attention has been given to the prevention, detection, and treatment for these patients. Herein, we discuss the latest understanding of the epidemiology, clinical characteristics, and therapeutic strategies of indeterminate CHB, to provide avenues for the management of these patients.

## Introduction

As a hepatotropic DNA virus, hepatitis B virus (HBV) can establish a persistent chronic infection, and cause a series of liver-related complications, including impaired liver function, fibrosis, cirrhosis, failure, and hepatocellular carcinoma (HCC) [1]. HBV is mainly transmitted by perinatal, percutaneous or sexual exposure, and by close person-to-person contact, among which perinatal transmission remains the most important cause of chronic infection [2]. The prevalence of HBV infection, as assessed by the presence of hepatitis B surface antigen (HBsAg), varies widely by regions [2]. Its morbidity and mortality have decreased owing to infant prophylaxis, early childhood vaccination, and medication to treat HBV infection; however, HBV has not yet been eradicated, mainly due to the lack of a virological cure for HBV infection [3–5]. Approximately 250 million people are living with HBV infection worldwide [3, 6], and a large proportion of liver fibrosis, cirrhosis, or HCC cases are associated with HBV infection [7]. Therefore, it is vital to understand the natural history of chronic hepatitis B (CHB) infection, and to investigate clinical preventive and therapeutic strategies to eliminate HBV.

According to the 2016 and 2018 Hepatitis B Guidelines proposed by the American Association for the Study of Liver Diseases (AASLD) [2, 4], CHB has been traditionally characterized into four immune phases, namely, the immune-tolerant phase, immune-active phase, inactive CHB phase, and immune reactivation phase, reflecting the dynamic correlations of HBV replication and evolution with host immune responses. Dynamic and serial monitoring of serum HBV antigens and antibodies, HBV DNA and alanine aminotransferase (ALT), as well as liver histopathology, helps to identify the immune phase of a chronic HBV infection [2, 4]. Patients with HBV infection can transition through different phases, and do not always evolve through these four phases in a subsequent manner [2, 4, 8, 9]. There is a high prevalence of HBV infection in China, and the Chinese Society of Hepatology, Chinese Medical Association [9, 10] has recommended that immune-active or reactivation CHB patients with elevated ALT levels should be treated with antiviral therapy; however, it may be controversial to treat CHB patients in immune-tolerant or inactive phase with HBsAg and HBV DNA positive and ALT beyond upper limits of normal (ULN). Furthermore, many CHB patients do not fit into any of these well-known phases, because their HBV DNA and/or ALT levels are outside of the defined ranges; these patients are considered “indeterminate patients” or in the “gray zone” [2, 4, 8]. The AASLD 2016 and 2018 HBV Guidelines [2, 4] suggest to dynamically monitor the serum HBV DNA and ALT levels of indeterminate patients rather than apply antiviral therapy. However, these patients may also be confronted with HBV-related complications [8, 11], and might benefit from antiviral therapy [12]. At present, there is no unified global definition of the indeterminate CHB phase. Although such a definition remains controversial, it is essential to reach a consensus on the management of indeterminate CHB patients, with accurate diagnostic and appropriate therapeutic strategies. This review discusses the epidemiology, clinical characteristics, and therapeutic strategies of indeterminate CHB to provide avenues for the management of these patients.

## Epidemiology

Although the incidence rates of new HBV infections have gradually decreased worldwide owing to infant prophylaxis, early childhood vaccination, and medication to treat HBV infection, the World Health Organization (WHO) has reported that there were still approximately 250 million (3.5%) people with chronic HBV infection, and more than 1 million deaths were attributable to CHB-related complications [3–6]. Specifically, the implementation of birth three-dose coverage of HBV vaccination, considered as one of the largest strides, is limited in many developing countries, which seems to be the largest constrain on eliminating HBV infection [3]. Besides, patients with HBV infection are not always evaluated and treated adequately [13]. Thus, the effective strategies to prevent HBV infection and CHB progression have not been fully put into effect [3]. Moreover, the uneven geographic distribution of HBV prevalence has been emphasized, and the majority of HBV infections concentrates in the Western Pacific Region (116 million people) and the African Region (81 million people), which is inversely proportional to the income level [13, 14]. For instance, the prevalence rate of HBsAg positivity is 5–6% in the whole population in China, and approximately 70 million people have chronic HBV infection, including 20–30 million CHB cases [10, 15]. Furthermore, according to data from the 2019 Global Burden of Disease (GBD) Study (https://vizhub.healthdata.org/gbd-results/), estimated 331,000 deaths were from HBV-related chronic liver diseases in 2019, which did not change significantly compared to the previous decade [13, 16]; if the current situation remains, the annual mortalities from CHB-related complications are expected to increase by 39% from 2015 to 2030 [13]. Of note, the mortalities from CHB in different regions vary worldwide, with an uneven geographic distribution analogous to HBV prevalence mentioned above [13, 14, 17]. Although WHO has raised a goal of eliminating viral hepatitis as a public health problem by 2030 [15], the current data indicate that HBV infection remains a major global health problem, especially in developing countries, and more attention should be given to CHB patients.

### The proportion of CHB patients with indeterminate phase

There have been considerably different epidemiological findings regarding indeterminate CHB patients [8, 18, 19]. One retrospective cohort study [8] recruited 3366 non-cirrhotic patients with untreated chronic HBV infection, and found that 38.7% of these patients were in indeterminate phase [19]; after a 10-year follow-up, 52.7% remained indeterminate, and 21.7% turned to the immune-active phase. Spradling PR et al. [11] demonstrated that more than half of 1598 CHB patients were indeterminate in a general US health care setting. Another retrospective study in China [18] that enrolled 4759 treatment-naïve CHB patients found that approximately 27.78% were defined as indeterminate (25.8%). Thus, the proportion of indeterminate patients has varied among studies based on distinct levels of upper/lower limits of normal (ULN/LLN) for HBsAg, HBV DNA, and ALT [2, 4, 10, 20]. These parameters are pivotal in the classification of immune phases for CHB [2, 4, 10, 20]. For instance, a study [21] of Asian Americans with CHB showed that 37% of these cases were indeterminate based on the conventional ULN for ALT (40 IU/L), whereas 33% were indeterminate when applying a modified ULN for ALT (30 IU/L for males and 19 IU/L for females). Although the epidemiological data of indeterminate patients may not be consistent in different studies due to different guidelines, the proportion of such CHB patients is not small and cannot be neglected, and more attention should be given to this population. A recent Chinese retrospective study [18] focused on untreated indeterminate CHB patients, and reported that indeterminate patients could be further divided into subgroups that were similar to the defined immune phases [2]. Specifically, 13.92% of indeterminate patients were similar to CHB patients with immune-tolerant phase but did not precisely fit into this phase; 7.79% were similar to CHB patients with immune-active phase, 24.73% were similar to patients with inactive phase, and 53.56% were similar to patients with reactivation phase [18]. Moreover, these subgroups of indeterminate patients showed differences regarding age and sex, suggesting that age and sex are crucial factors that may affect the distribution of indeterminate patients [18, 22].

### Poor prognosis of CHB patients with indeterminate phase

**Fig. 1:**
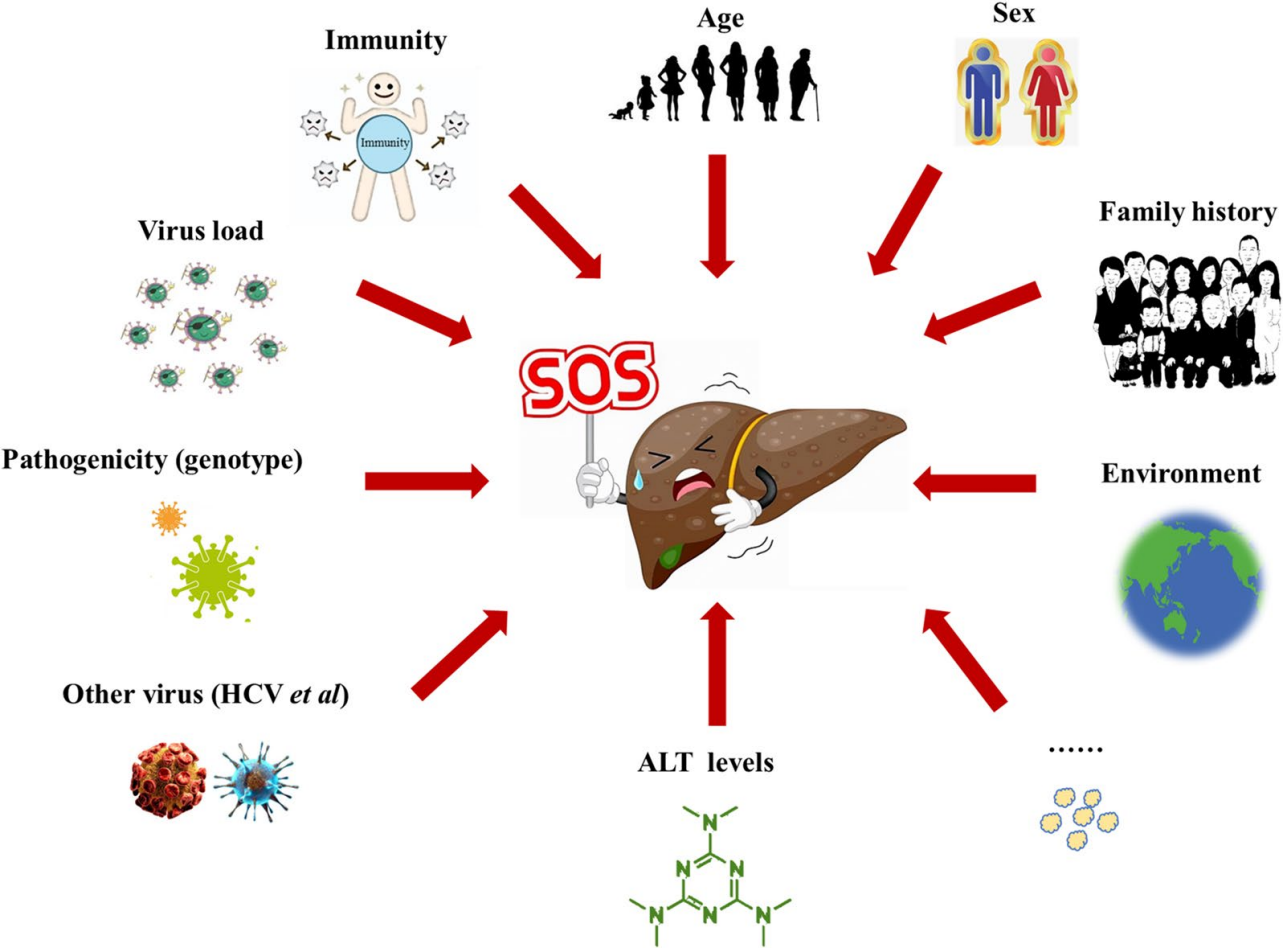
Risk factors of disease progression of CHB patients with indeterminate phase. CHB: chronic hepatitis B

The current evidence suggests that indeterminate CHB patients tend to have a poor prognosis [23]. Spradling et al. [11] found that 9% of indeterminate patients developed liver cirrhosis in 6.3 years, which was three times higher than CHB patients in immune inactive phase. Consistent with the above finding, Huang et al. [8] indicated that CHB patients who remained indeterminate had a higher 10-year cumulative HCC incidence than CHB patients who remained immune inactive. Moreover, hepatitis B e antigen (HBeAg)-negative indeterminate patients with serum HBV DNA levels ≥ 2 × 10^3^ IU/mL and normal ALT levels (≤ ULN, 40 U/L) were found to have remarkably higher risks of HBV-related liver diseases, such as necroinflammation (≥ G2) and fibrosis (≥ F2), than patients in immune inactive phase (HBV DNA < 2 × 10^3^ IU/mL) [24, 25]. Yao KF et al. [18] also reported that higher proportions of indeterminate CHB patients may experience liver fibrosis or even cirrhosis. Some factors, such as age, sex, a family history of HCC, virus load and genotype, may affect disease progression in indeterminate patients (Fig. 1) [4, 12, 18]. For instance, Huang DQ et al. [8] showed that age was independently related to HCC development and was nine times higher in indeterminate patients over 40 years, and 18.4 times higher for those older than 45, indicating that older age could be considered as an independent risk factor for other advanced liver diseases in indeterminate patients [8, 18]. Besides, the HBV genotype may also play an important role in the progression of HBV-related complications and in the therapeutic efficacy [2].

Therefore, more work is needed to clarify the global epidemiology of indeterminate CHB patients, and more attention should be given to these patients with regard to differences in age, sex, family history, race and geographic location.

### Laboratory evaluations for chronic HBV infection

It is necessary to determine the status of a chronic HBV infection and any liver-related complications, which are crucial to guiding the therapeutic strategy [10]. Thus, novel biomarkers with good accuracy should be explored and thoroughly investigated to facilitate the screening, diagnosis, and prognosis of chronic HBV infection.

### Laboratory evaluations of HBV

The covalently closed circular DNA (cccDNA) of HBV in hepatocytes is the main cause of persistent infection, and is an accurate indicator of HBV presence in the body [26]. HBV cccDNA is the only known template for pregenomic RNA transcription, which produces the template for reverse transcription and viral genome replication [5]. Thus, monitoring intrahepatic cccDNA is important for deciding an antiviral therapeutic strategy [27]; however, certain limitations exist. Specifically, HBV cccDNA is mainly located in the nucleus of infected hepatocytes; thus, its detection is complicated, because it requires invasive liver biopsy, and there can be interobserver variability as well [28, 29]. In addition, there is no standardized assay for cccDNA quantification, but quantitative PCR methods are being standardized [5].

#### Serum markers of cccDNA: HBV DNA

To address this problem, researchers have developed various non-invasive tests to assess HBV cccDNA levels and transcriptional activity, among which the most classic is serum HBV DNA quantitation [2, 10]. Serum HBV DNA is used to estimate viral replication, and guide decisions about antiviral therapeutic strategies [10, 30]. However, the detection results of HBV DNA levels are not consistent among the various available kits because of differences in the primers and reagents. Additionally, some CHB patients showed different levels after nucleos(t)ide analog (NA) treatment [31].

#### Serum markers of cccDNA: HBsAg

HBsAg is another potential biomarker, and is expressed by HBV cccDNA and viral DNA that has integrated into the host genome in infected hepatocytes [10, 27, 32–35]. HBsAg is correlated with HBV transcriptional activity, serum HBV DNA levels, and HBeAg status [35, 36]. The quantitative detection of serum HBsAg levels may help to distinguish patients among the different phases of chronic HBV infection [35–39]. Notably, HBsAg consists of three kinds of proteins, which are large (L), middle (M), and small (S) HBs. The ratio of LHBs and MHBs was considered as a better predictors of HBeAg-negative chronic HBV infection and HBeAg-negative CHB than the total HBsAg concentration [40].

#### Serum markers of cccDNA: hepatitis B core-related antigen (HBcrAg)

HBcrAg is also considered a useful and sensitive biomarker that can provide evidence for intrahepatic HBV cccDNA [41, 42]. Chen EQ et al. [42] identified a significant correlation between serum HBcrAg and intrahepatic HBV cccDNA, and this correlation was stronger than that of HBV cccDNA with serum HBsAg or HBV DNA. Further analysis showed an association between decreased HBV cccDNA and decreased serum HBsAg or HBcrAg. The serum HBcrAg level could reflect the presence and transcriptional activity of intrahepatic cccDNA in CHB patients, while it could not indicate the transcriptional activity of integrated HBV DNA in infected hepatocytes [34]. However, the correlations between HBcrAg and HBV RNA are unclear. Moreover, quantitative detection of serum HBcrAg levels may help determine the natural history of chronic HBV infection, and accurately predict spontaneous HBeAg seroconversion in CHB patients [42]. Specifically, serum HBcrAg levels are higher in immune-tolerant and immune-active phases, and lower in inactive and reactivation phases [43]. Furthermore, HBcrAg has been proven to be an effective indicator of the prognosis and antiviral therapeutic efficacy in CHB patients [44–46]. For instance, serum HBcrAg levels may be used to stratify HCC risk in CHB patients with indeterminate phase [47–49]. A study, enrolled two retrospective cohorts in Taiwan and Japan, reported that serum HBcrAg level of 10,000 U/mL could be an effective cut-off value for HCC risk stratification in HBeAg-negative CHB patients with indeterminate phase; and the 10-year HCC cumulative incidence was 5.33% in patients with high serum HBcrAg levels, which was significantly higher than that of 0.51% in patients with lower levels [49]. After that, Tseng TC et al. showed that HBcrAg-based score may be better than HBV DNA-based score to predict HCC risks in indeterminate CHB patients who are HBeAg-negative [50]. Another follow-up study also indicated that serum HBcrAg was better than HBV DNA and HBeAg in predicting HCC occurrence, and an HBcrAg level > 2.9 log U/mL was an independent predictor of HCC incidence [47]. In addition, Cheung et al. [48] found that pretreatment (NA) HBcrAg levels were significantly higher in an HCC group than in a non-HCC group, suggesting that pretreatment HBcrAg > 47.1 kU/mL independently predicted HCC development in CHB patients (OR 3.29, 95% CI 1.66–6.52). Of note, serum HBcrAg could also predict HCC recurrence after resection or radiofrequency ablation [46]. Therefore, serum HBcrAg may become a newly recognized biomarker for monitoring disease states, evaluating therapeutic efficacy and drug withdrawal, and predicting HCC development or recurrence [44–48, 51, 52]. Nevertheless, the optimal cut-off values of serum HBcrAg for the defined immune phases and indeterminate phase remain to be determined.

#### Serum markers of cccDNA: serum HBV RNA

In recent years, serum HBV RNA has been discovered and recognized as an HBV virological indicator [53–55]. Although serum HBV RNA levels vary during the natural phases of chronic HBV infection, the distribution pattern is similar to that of serum HBV DNA among the different immune phases, suggesting a predictive effect on the immune phases of chronic HBV infection [27]. Serum HBV RNA levels can also reflect the concentration and transcriptional activity of cccDNA in hepatocytes [56–58], and may have special significance in guiding NA administration [55, 59]. In contrast, some studies have indicated that serum HBV RNA does not have notable advantages in distinguishing the immune phases in CHB patients, compared with other traditional biomarkers [10]. Unfortunately, there is no standard or standardized method for the quantitative detection of HBV RNA; therefore, its standardization and traceability need to be clarified.

In summary, additional longitudinal studies with larger sample sizes are needed to further investigate the clinical utility of these biomarkers.

### Laboratory evaluations of liver histopathology

**Fig. 2:**
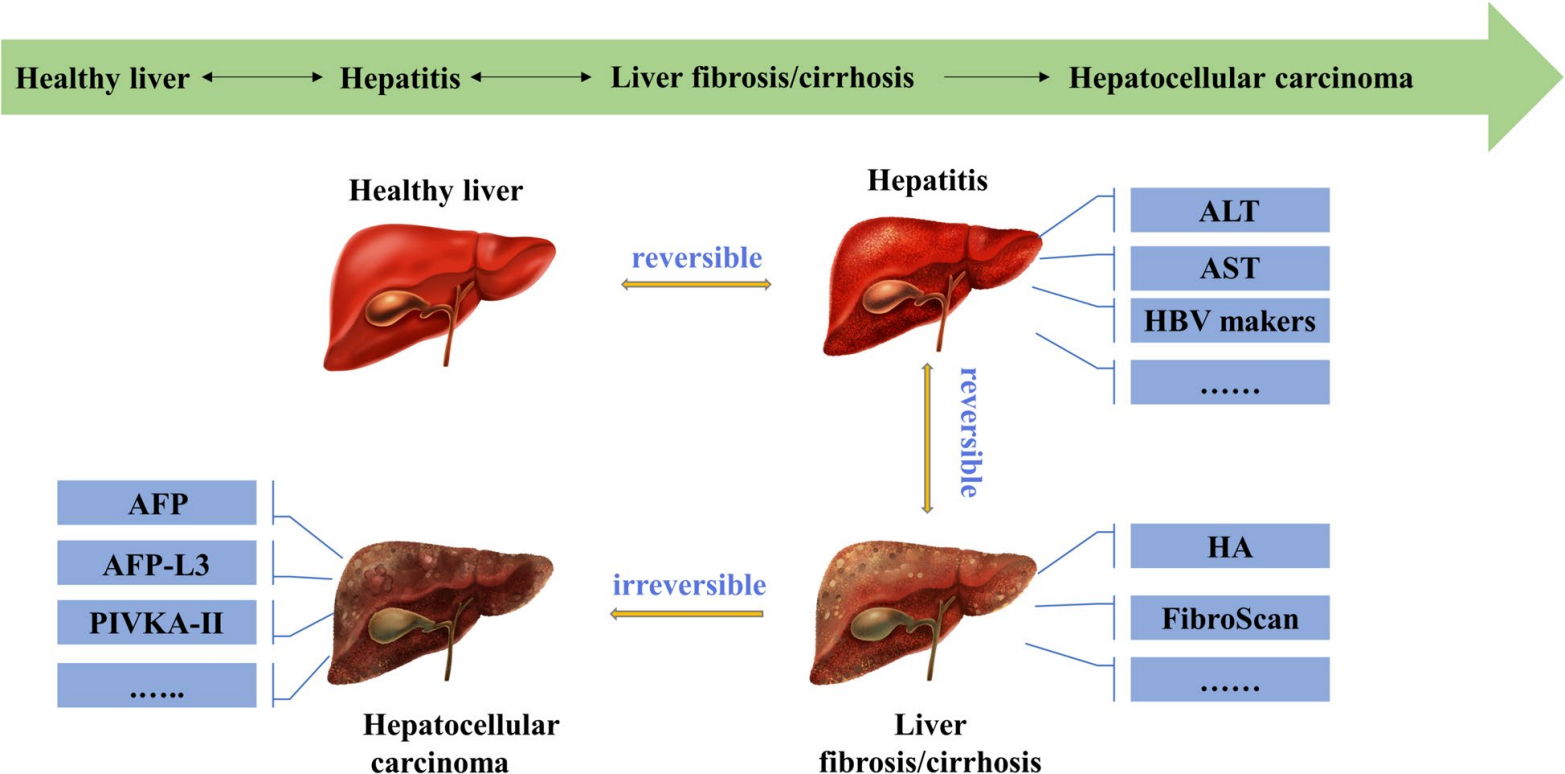
Liver histopathology and relevant serum markers. ALT: alanine aminotransferase; AST: aspartate aminotransferase; HBV: hepatitis B virus; HA: hyaluronidase; AFP: alpha-fetoprotein; AFP-L3: Lens culinaris agglutinin-reactive fraction of alpha-fetoprotein; PIVKA-II: prothrombin induced by vitamin K deficiency or antagonist-II

For CHB patients, liver biopsy is regarded as the gold standard to assess the severity of liver inflammation, fibrosis, or cirrhosis [10]; however, patients are reluctant to have repeated biopsies to monitor disease progression [60] due to its invasiveness and risk of complications [61]. Non-invasive tests may contribute to evaluating liver histopathology (Fig. 2); these tests include serum alanine aminotransferase (ALT), aspartate aminotransferase (AST), albumin, prothrombin time (PT), the AST-to-platelet ratio index (APRI), hyaluronidase (HA), laminin (LN), and N-terminal propeptide of collagen type I (PINP) [4, 62, 63]. ALT and AST are regarded as standard enzymes used to evaluate the degree of hepatocyte damage, and serum ALT detection is a sensitive indicator for the diagnosis of viral hepatitis [64]. The following is a brief description of these biomarkers of liver histopathology.

#### Serum markers of fibrosis and cirrhosis

As a high-molecular-weight glycosaminoglycan that is found in the extracellular matrix (ECM), HA could enter the circulation during the ECM turnover process, and elevated HA levels in circulation may indicate increased production of HA or reduced clearance of circulating HA, thus may correlate with liver inflammation and fibrosis [62]. Therefore, HA is considered as a sensitive and specific indicator of liver fibrosis among the various biochemical indicators, and is also an effective indicator of disease progression in CHB patients [62]. Transient elastography (FibroScan) [65] has also been used to the diagnose liver fibrosis with high accuracy, although it cannot provide information about intrahepatic inflammation. Additionally, thrombocytopenia has been suggested as a surrogate of liver cirrhosis and as an HCC predictor in patients with viral hepatitis [66].

#### Serum markers of HCC

Although tumor biomarkers, including alpha-fetoprotein (AFP), *Lens culinaris agglutinin*-reactive fraction of alpha-fetoprotein (AFP-L3) and prothrombin induced by vitamin K deficiency or antagonist-II (PIVKA-II), could not be used to directly diagnose HCC, they may predict tumor progression and even outcomes of HCC patients [67]. Since AFP was indicated in serum of HCC patients in 1964, it is considered as the primary biomarkers for HCC [68]. As an alpha globulin containing glycoprotein, the expression of AFP is increased during pregnancy by fetal liver and decreased to trace level after childbirth within less than 1 month [69]. High serum AFP level is usually associated with the presence and development of HCC [68, 70]. However, the elevation of serum AFP levels also exists in non-HCC diseases, such as hepatitis, cholangiocarcinoma, testicular germ cell tumor, and metastatic colon cancer [70]. With a sensitivity of 18–60% and a specificity of 85–90%, AFP alone is not recommended for HCC screening [70]. Based on its binding capacity to *lectin lens culinaris* agglutinin, AFP could be classified as three subtypes which are AFP-L1, AFP-L2, and AFP-L3 [69]. AFP-L1 increases in chronic hepatitis and liver cirrhosis, and AFP-L2 is increased in yolk sac tumors and may be detected in pregnant women [71]. With a higher specificity of 92.0–99.4%, AFP-L3 is regarded as a more specific biomarker for HCC [72, 73]; however, it has a low sensitivity ranging from 18.8 to 37% for HCC, and may be not relevant to HCC with a total AFP level lower than 20 ng/mL [72–74]. In a word, the concentrations of AFP and AFP-L3 should be detected together to facilitate early diagnosis of HCC [75]. Also known as Des-γ-carboxyprothrombin, PIVKA-II was considered as one of the tumor-related biomarkers [71, 76]. With a higher sensitivity of 72.7% and a specificity of 90% for HCC, the elevated serum PIVKA-II levels was not closely related to non-HCC liver diseases compared to serum AFP levels [70, 77]. It was reported that significant correlations existed between serum PIVKA-II levels and HCC clinicopathological characteristics, such as tumor size and TNM stage [76], and the performance of PIVKA-II plus AFP for HCC identification was superior to each of these biomarkers used alone [78]. Besides, PIVKA-II may improve the identification of patients with AFP-negative HCC [74]. Additionally, dickkopf-1 and circulating IgG are also regarded as novel promising biomarkers for HCC [79–83]. Altogether, combinations of these biomarkers might provide better prospects in clinical applications.

#### Laboratory evaluations of liver histopathology in indeterminate CHB patients

To more conveniently and accurately evaluate liver histopathology in indeterminate CHB patients, Pan AN et al. developed a new scoring system called the “Significant Histological Model (SHM) scoring system”, which could predict liver histopathological changes in indeterminate patients [84]. The SHM scoring system suggests that AST, platelet (PLT) counts, albumin, and HBV DNA (log_10_ IU/mL) are independent predictors of liver histological changes in indeterminate CHB patients. The model [84] showed good accuracy in identifying indeterminate patients with/without liver histological changes (logistic y = 3.339 + 0.06 × AST−0.06 × PLT−0.068 × albumin−0.246 × HBV DNA [log_10_ IU/mL]). Other non-invasive indicators, such as the APRI, Lok index, Forn index, FIB-4, and Zeng score, could not accurately predict the degree of liver fibrosis [63, 85–88]. The SHM scoring system has not been fully evaluated or directly compared with other non-invasive indicators, and its clinical application requires further investigation [84].

Although these indicators may have limited accuracies in identifying CHB patients with liver fibrosis, they may play crucial and guiding roles in decision-making within disease staging and therapeutic strategy selection [4]. However, the associations among these indicators should be thoroughly investigated to identify a more effective system that would facilitate the diagnosis and prognosis of indeterminate CHB patients.

### Clinical characteristics of indeterminate CHB patients

The interactions of HBV with the host and environmental factors are complicated [8, 12, 24, 89]. To our knowledge, HBV is not directly cytopathic, and host immune responses to HBV-infected hepatocytes are thought to mediate liver injury and the development of cirrhosis and HCC [2]. In addition, persistent or recurrent liver inflammation and incomplete HBV clearance might contribute to CHB development [10]. After primary HBV infection, the body initiates a nonspecific immune response, followed by a specific immune response [10]. Adult-acquired HBV is generally cleared by the host immune system, while chronic HBV infection is usually acquired from perinatal or horizontal infection [90]. Since chronic HBV infection is dynamic, it is necessary to regularly detect the levels of serum HBeAg, HBV DNA, and ALT [4, 10]. According to the liver disease severity, host immune response, and the natural history of chronic HBV infection, patients with chronic HBV infection are classified into four immune phases [2, 4, 10], but many CHB patients do not fit into any of these defined phases [2]. The guidelines [2, 4, 20] proposed by AASLD and the European Association for the Study of the Liver (EASL) have officially designated “indeterminate” gray zone (GZ), which indicate that the patient’s HBV DNA and ALT levels do not fall into the same phase; serial monitoring of the serum HBeAg, HBV DNA, and ALT levels is suggested in most instances, even after a complete assessment is conducted. A Chinese study [18] subdivided indeterminate CHB patients into four groups based on the following defined phases [2]: ① GZ-A: HBeAg positive, normal serum ALT and HBV DNA ≤ 10^6^ IU/mL; ② GZ-B: HBeAg positive, elevated serum ALT and HBV DNA ≤ 2 × 10^4^ IU/mL; ③ GZ-C: HBeAg negative, normal ALT and HBV DNA ≥ 2 × 10^3^ IU/mL; and ④ GZ-D: HBeAg negative, elevated serum ALT and HBV DNA ≤ 2 × 10^3^ IU/mL. To date, there is no unified definition of indeterminate CHB; therefore, this patient population needs to be further elucidated [2].

### Immune-tolerant phase and indeterminate phase

Also known as HBeAg-positive chronic HBV infection [10, 20], the immune-tolerant phase is usually regarded as a benign disease course [91], and is characterized by high levels of serum HBsAg and HBV DNA, normal or minimally elevated ALT, and minor or no necroinflammation or fibrosis in the liver [2, 10, 92]. Different countries have distinct definitions of the immune-tolerant phase regarding the serum HBV DNA level. The diagnostic values of HBV DNA are > 2 × 10^7^ IU/mL in China [10], > 1 × 10^7^ IU/mL in European countries [20], > 1 × 10^6^ IU/mL in the United States [2], and > 2 × 10^4^ IU/mL according to the Asia–Pacific Guidelines [93]. Therefore, the proportions of indeterminate CHB patients with immune tolerance are quite different according to these distinct guidelines. Specifically, indeterminate CHB patients are characterized by HBeAg and HBsAg positivity, HBV DNA ≤ 2 × 10^4^ IU/mL, and normal serum ALT levels (≤ 35 U/L for men and 25 U/L for women) in accordance with the AASLD Guidelines [2].

The underlying mechanisms of immune tolerance are mainly described as follows: ① Persistence of cccDNA. Upon infection, HBV nucleic acid enters the hepatocellular nucleus, and forms a primitive template for viral replication, namely, cccDNA. It remains in the nucleus “wrapped” by histones, which act as the virus template being copied and transcribed in the nucleus, and then generates new HBV particles causing persistent infection [28]. Although the current antiviral medications are designed to inhibit HBV DNA replication, it is difficult to target intrahepatic cccDNA. In addition, HBV cccDNA has high stability and a long half-life, which could also explain the difficulty of HBV clearance [26]. ② High variability of the HBV gene. The HBsAg antigenicity or serum concentrations could be changed after mutations of the pre-S/S region [94, 95], which may help the virus to escape neutralization by the corresponding antibodies and induce T cells to develop immune tolerance to the target antigen. ③ Presence of cellular immunity. Cellular immunity and the production of cytokines related to this process may play important roles in mediating host immune tolerance [93, 96, 97]. ④ Polymorphism of host genes. Some studies [98, 99] have shown that host gene polymorphisms are associated with immune tolerance. For instance, HLA-DPA1 and HLA-DPB are protective against chronic HBV infection in Asian populations [98], while rs7453920-G(HLA-DQ) and rs2856718-A(HLA-DQ) are associated with chronic HBV infection [99]. During the tolerant phase, HBV remains quiescent for several weeks during which the host immune system does not respond to the HBV infection [100]. Some researchers have suggested changing the “immune-tolerant phase” to the “high replication low inflammation period (HRLI)” due to the absence of immunological evidence [92]. However, there is no sufficient evidence to rename this phase [100, 101], but the EASL 2017 Guidelines [20] use the term “HBeAg-positive chronic HBV infection” based on the serum HBeAg status. Nevertheless, no specific definition has been recognized worldwide to replace the term “immune-tolerant phase”.

### Immune-active phase and indeterminate phase

Not all CHB patients go through the four phases in order, for instance, most patients who were infected with HBV in adolescence or adulthood may directly enter the immune clearance phase rather than the immune tolerance phase, and the perinatally or early childhood-acquired chronic HBV may have a long immune-tolerant phase [9, 102]. During the immune-active phase, CHB patients are considered to have HBeAg-positive chronic hepatitis B [20], and are characterized by the presence of serum HBeAg, elevated serum HBV DNA, and intermittently or persistently elevated serum ALT, in conjunction with chronic moderate-to-severe necroinflammation or fibrosis [2, 4]. In this phase, host immune tolerance to HBV is lost, and the immune system attacks HBV-infected hepatocytes, resulting in decreased HBV DNA levels and elevated ALT levels. However, some CHB patients with similar characteristics fall outside the above ranges, and are considered indeterminate. For instance, indeterminate CHB patients are usually characterized by HBeAg and HBsAg positivity, HBV DNA positivity (between 2 × 10^3^ IU/mL and 2 × 10^4^ IU/mL), and continuously or repeatedly abnormal ALT levels (≤ 2 ULN) [2].

### Immune inactive phase and indeterminate phase

The immune inactive phase, previously the inactive carrier phase [4, 103], is known as HBeAg-negative chronic HBV infection [20], and is characterized by the presence of HBeAg seroconversion, low or undetectable HBV DNA levels, persistently normal ALT levels, and minimal necroinflammation and variable fibrosis in the liver [2, 4, 10]. Similarly, some HBeAg-negative CHB patients are considered indeterminate, with serum HBV DNA levels ≥ 2 × 10^3^ IU/mL and normal ALT levels, or HBV DNA levels < 2 × 10^3^ IU/mL and elevated serum ALT levels [2].

### Immune reactivation phase and indeterminate phase

Spontaneously or subsequent to antiviral therapy [104], some CHB patients enter the reactivation phase, which is also called HBeAg-negative chronic hepatitis B [2, 10, 20]. The immune reactivation phase is characterized by antibody to hepatitis B e antigen (anti-HBe) positivity, elevated HBV DNA levels, fluctuating or persistently elevated ALT, and moderate-to-severe necroinflammation or fibrosis (≥ G2/S2) [2, 4, 10, 20]. The EASL 2012 and 2017 Clinical Practice Guidelines [20, 105] state that CHB patients in reactivation phase usually have low rates of spontaneous disease remission. Some patients do not meet the diagnostic criteria of the other three defined phases, but do not fit into the reactivation phase due to one of the above indicators being out of range. These indeterminate CHB patients are usually characterized by low levels of HBV DNA (< 2 × 10^3^ IU/mL) and abnormal ALT levels (≥ 2 ULN), or HBV DNA ≥ 2 × 10^3^ IU/mL and slightly elevated ALT levels (< 2 ULN) [2].

### Therapeutic strategies for indeterminate CHB patients

The EASL 2017 Guidelines on the management of HBV infection and the AASLD 2018 Hepatitis B Guidelines have proposed the optimal goal of CHB treatment, which is to suppress HBV replication in a sustained manner and even eliminate the virus from infected hepatocytes to prevent disease progression, resulting in longer survival and improved quality of life [2, 20]. Afterwards, the Chinese Medical Association has proposed a comprehensive and dynamic assessment for chronic HBV infection [10], including serum HBV DNA and ALT levels, liver function and disease severity, age, family history of HCC, and concomitant diseases, to guide clinical decisions and therapeutic strategies [4, 106].

### Clinical treatment of HBsAg-positive and HBeAg-positive CHB patients in immune-tolerant, immune-active, or indeterminate phase

The immune-tolerant phase is characterized by a higher HBV load without obvious liver histopathological changes, indicating a "peaceful coexistence" of HBV and the host [107, 108]. Most HBV-related guidelines recommend to not treat these patients with antiviral therapy [2, 4, 10, 100, 109, 110]. Different guidelines have distinct diagnostic values of serum indicators for staging CHB patients. For example, the AASLD 2018 Guidelines [2] state that immune-tolerant and indeterminate CHB patients [2] with HBV DNA levels > 2 × 10^4^ IU/mL and ALT ≤ ULN should not be treated with antiviral therapy; in addition, it is necessary to monitor the HBV DNA and ALT levels every 3–6 months and HBeAg every 6–12 months. Notably, serum ALT levels have no direct correlation with the liver viral load; however, serum ALT levels within the normal range could not indicate no or slight inflammatory activity in the liver either [111, 112]. A multinational systematic analysis [112] of 830 CHB patients with normal serum ALT levels found that 20.7% were in the severe liver fibrosis stage (≥ F2), suggesting that serum ALT levels did not accurately reflect the liver histopathology. A meta-analysis and systematic review [113] indicated that CHB patients with normal or slightly elevated serum ALT levels may also have HBV-related complications, and could benefit from antiviral therapy. Additionally, a study [114] conducted in South Korea found that the cumulative incidence of HCC in untreated immune-tolerant patients (ALT < 30 U/L for males, ALT < 19 U/L for females) was 12.7%, which was significantly higher than that in treated patients in immune reactivation phase (6.1%, ALT > 80 U/L). Obviously, these so-called immune-tolerant patients should be treated with antiviral medication. The AASLD HBV Guidelines [2, 4] suggest antiviral therapy for indeterminate CHB patients over 40 years old with normal ALT, elevated HBV DNA and liver biopsy showing moderate-to-severe necroinflammation or fibrosis. Some indeterminate CHB patients are characterized by mildly elevated serum ALT levels (< 2 ULN) and HBV DNA levels (> 2 × 10^4^ IU/mL) [2]. For these patients, other causes of ALT elevation should be excluded, and an assessment of disease severity should be performed with non-invasive tests and/or liver biopsy [2]. If liver histopathological changes indicate ≥ F2 or ≥ A3 and slightly elevated ALT (< 2 ULN) persists, these indeterminate patients should be treated with antiviral therapy, especially if they are over 40 years old [2]. For indeterminate patients with HBV DNA levels between 2 × 10^3^ IU/mL and 2 × 10^4^ IU/mL, regular monitoring should be performed every 1–3 months; if HBV DNA persists for > 6 months, antiviral therapy should be considered, regardless of the serum ALT level [2].

For CHB patients in either immune-tolerant or indeterminate phase, comprehensive and dynamic assessments should be performed to evaluate whether they have real immune tolerance and have any intra- or extra-liver complications. Other factors, such as age, sex, and family history of HCC, should be considered in this process [106].

The immune-active phase is believed to be the best time for antiviral therapy to achieve satisfactory therapeutic efficacy with decreased risk of liver-related complications [2, 4, 10]. In this phase, HBV is actively replicating, and the host’s immune system is also activated; hence, HBV can be recognized and attacked by the immune system, but the patient may experience recurrent abnormal liver function [10]. Therefore, it is recommended to treat immune-active patients with antiviral therapy [10]. For certain indeterminate patients with HBV DNA levels < 2 × 10^4^ IU/mL and elevated ALT (≥ 2 ULN), regular monitoring of HBeAg, HBV DNA, and ALT should be performed, as well as non-invasive tests and/or biopsy for liver histopathological changes [2]. These indeterminate patients (with elevated ALT and HBV DNA levels between 2 × 10^3^ IU/mL and 2 × 10^4^ IU/mL) should be treated with antiviral therapy, especially if they are older than 40 years, or have liver cirrhosis, a family history of HCC, previous treatment history, extrahepatic manifestations, or a long duration of HBV infection [2, 4].

### Clinical treatment of HBsAg-positive and HBeAg-negative CHB patients in immune inactive, reactivation phases, or indeterminate phase

Theoretically, immune inactive patients usually have low serum HBsAg levels, undetectable or low levels of HBV DNA (< 2 × 10^3^ IU/mL), and persistently normal ALT levels [2, 10]; however, some patients with immune inactivation have higher HBV DNA levels or slightly elevated ATL levels (< 2 ULN), and are considered indeterminate [2]. The AASLD Hepatitis B Guidance [2, 4] suggests that antiviral therapy is generally not recommended for immune inactive patients, and regular monitoring of the HBV DNA and ALT levels is recommended every 3 ~ 6 months, as well as HBsAg annually. Some researchers believe that immune inactive patients should not be treated with antiviral therapy for the following reasons: ① There is a lower risk of disease progression. For example, a study [108] of 361 immune inactive CHB patients showed that only 2.8% experienced disease progression within a 4-year follow-up. A similar study by Tong MJ et al. [115] recruited 146 HBeAg-negative CHB patients with normal ALT levels and HBV DNA ≤ 10 × 10^3^ IU/mL and followed them for 8 ± 6.3 years; none of the patients progressed to liver cirrhosis, and only 2 developed HCC. Bonacci M et al. [12] found that the proportion of HBeAg-negative patients among indeterminate CHB patients was only 6.3%, and none developed liver fibrosis or cirrhosis during an 8.2-year follow-up. The prognosis of immune inactive patients is favorable, and transiently elevated ALT and HBV DNA levels may have minimal clinical significance [115]. ② Economic burden. Once started, antiviral therapy usually lasts for 5–10 years, and can even last a lifetime, which can result in a large economic burden on these patients and their families [116]. And Zhang H et al. [117] have already revealed the economic burden on CHB patients in China. ③ Adverse effects of antiviral therapy. Kwon JH et al. [118] and Buti M et al. [119] found that some patients develop renal dysfunction with different severities after 7 years of antiviral therapy with tenofovir, suggesting cautious consideration of antiviral strategies for patients with chronic HBV infection. However, whether immune inactive patients should be treated with antiviral therapy remains controversial. Duan MH et al. [24] found that ALT > 20 U/L was a good independent predictive factor for evaluating liver histopathology for immune inactive CHB patients, and liver biopsy or non-invasive methods should be performed to evaluate the liver histopathological changes to make decisions about antiviral therapy [2]. Older (> 40 years) male CHB patients with delayed HBeAg seroconversion and a family history of liver cirrhosis or HCC may have significantly higher risks of clinical events [120, 121]. Thereafter, the EASL 2017 Guidelines [20] and the China 2019 Guidelines [10] recommend antiviral therapy for immune inactive patients with a family history of liver cirrhosis or cancer, regardless of liver histopathology.

Different clinical studies have reported different risks of disease progression in HBeAg-negative indeterminate patients. For example, Spradling PR et al. [11] conducted a large cohort study of CHB patients from 2006 to 2013, and found that 9% of indeterminate patients progressed to liver cirrhosis, and indeterminate patients with similar immune inactive characteristics may have a higher risk of disease progression than defined immune inactive patients. Moreover, the HBV DNA load was found to be an independent risk factor for related clinical events in HBeAg-negative CHB patients [122]. In addition, the proportion of patients with liver necrotizing inflammation and fibrosis was significantly higher in HBeAg-negative indeterminate patients with normal ALT levels (≤ ULN, 40 U/L) and HBV DNA levels ≥ 2 × 10^3^ IU/mL than in immune inactive patients with normal ALT and HBV DNA levels < 2 × 10^3^ [24, 25]. A large clinical and community study from the United States and Taiwan, China found that antiviral therapy for patients with HBV DNA ≥ 2 × 10^3^ IU/mL reduced the risk of HCC by 77%, regardless of HBeAg, ALT, sex, age, and liver cirrhosis status or treatment medication [123]. However, a few studies have focused on antiviral therapy for immune inactive patients and the related indeterminate phase, and more studies are needed to provide evidence-based data to facilitate the clinical application of antiviral therapy. According to the AASLD guidelines [2], for indeterminate patients with HBV DNA levels ≥ 2 × 10^3^ IU/mL and normal ALT levels, regular monitoring should be performed every 3 months for 1 year and every 6 months thereafter. For indeterminate patients with slightly elevated ALT levels (≤ 2 ULN), regardless of HBV DNA levels, other causes of ALT elevation should be excluded, and an assessment of disease severity should be performed with non-invasive tests and/or biopsy for liver histopathological changes [2]. If liver histopathological changes indicate ≥ F2 or ≥ A3, antiviral therapy should be performed; if slightly elevated ALT levels (> ULN) with HBV DNA ≥ 2 × 10^3^ IU/mL persist, these indeterminate patients should be treated with antiviral therapy, especially if they are over 40 years old [2].

Antiviral therapy is also recommended for patients in immune reactivation phase [2, 4, 10]. According to the AASLD guidelines [2], indeterminate patients with HBV DNA levels < 2 × 10^3^ IU/mL and elevated ALT (≥ 2 ULN) should undergo regular monitoring of HBeAg, HBV DNA and ALT, as well as non-invasive tests and/or biopsy for liver histopathological changes.

### Future potential of therapeutic expansion for indeterminate CHB

It is worth noting that there is still no international consensus to guide the management of indeterminate CHB patients, and some differences exist among different guidelines. The AASLD 2016 and 2018 Hepatitis B Guidelines [2, 4] suggest to dynamically monitor the serum HBV DNA and ALT levels in indeterminate patients rather than apply antiviral therapy. While, the Chinese Expert Opinion on expanding anti-HBV treatment for CHB recommends to start antiviral therapy for untreated indeterminate patients with uncertain HBV DNA and ALT patterns after 1-year follow-up [124]. Certain studies indicate that indeterminate CHB patients may be confronted with HBV-related complications, and propose antiviral therapy to prevent disease progression and reduce HCC risks [8, 11, 12, 23–25, 125]. For instance, a randomized, double-blind, placebo-controlled study conducted in Taiwan, China showed that tenofovir disoproxil fumarate could reduce the risk of liver fibrosis in patients with non-cirrhotic CHB and minimally raised ALT [126]. Moreover, another multi-center study conducted in 14 centers in U.S., Europe and Asia reported that HCC risk in CHB is higher in indeterminate phase compared to the immune inactive phase, and antiviral therapy could reduce HCC risk by 70% among indeterminate CHB patients without advanced fibrosis [127]. In a word, expanding antiviral therapy may benefit indeterminate CHB patients from preventing disease progression, which is of great significance.

## Conclusion

Chronic HBV infection is a major health problem worldwide. One of the main issues for this infection is to decide which patients will benefit from antiviral therapy. The precise classification of the immune phases of CHB patients could help in evaluating disease prognosis and developing therapeutic strategies [10, 128]. However, certain CHB patients do not fit into any of the defined phases, and are considered to be in an “indeterminate” gray zone [4]. Although the distributions of indeterminate CHB patients vary due to distinct epidemiological data and classification strategies, approximately 30% ~ 40% of CHB patients are in indeterminate phase [2], and the proportions of indeterminate patients with distinct characteristics can vary [18]. Current evidence has shown that the prognosis of indeterminate patients is not optimistic, and they also have a higher risk of disease progression to liver cirrhosis or HCC. Factors, such as age, male sex, HBeAg positivity, higher HBV DNA or ALT levels, lower albumin levels or PLT counts, and a family history of HCC, have been associated with disease progression [8, 18, 122]. Accurate diagnosis is crucial to establish the immune phase and choose the optimal therapeutic strategy. Novel efficient assays should be investigated and verified. Furthermore, the therapeutic strategy for indeterminate CHB patients remains controversial. Clinicians should make a comprehensive assessment of these patients, based on the virology and immunology indicators, imaging examination, age, sex, and a family history of HCC. Regular monitoring of these patients should be performed, and antiviral therapy should be given to patients who are at high risk of disease progression. Although many advances have been made in providing therapeutic strategies for indeterminate CHB patients, there is still a long way to go to improve the prediction, detection, therapeutic timing, scheme, course, efficacy, and prognosis in this patient population. Multi-center and multi-country cohort clinical trials with large numbers of indeterminate CHB patients should be launched.

## Data Availability

Not applicable.
